# Get Beyond Limits: From Colloidal Tectonics Concept to the Engineering of Eco-Friendly Catalytic Systems

**DOI:** 10.3389/fchem.2018.00168

**Published:** 2018-05-25

**Authors:** Loïc Leclercq

**Affiliations:** Université de Lille, Faculté des Sciences et Technologies, Unité de Catalyse et Chimie du Solide, UMR Centre National de la Recherche Scientifique 8181, Equipe CÏSCO, Lille, France

**Keywords:** colloidal tectonics, attractive forces, nanostructured arrangements, supracolloids, dispersions, pickering emulsions, catalytic colloidal systems, green chemistry

## Abstract

The interactions between two or more molecules or colloidal particles can be used to obtain a variety of self-assembled systems called supramolecules or supracolloids. There is a clear, but neglected, convergence between these two fields. Indeed, the packing of molecules into colloidal or supracolloidal particles emerges as a smart solution to build an infinite variety of reversible systems with predictable properties. In this respect, the molecular building blocks are called “tectons” whereas “colloidal tectonics” describes the spontaneous formation of (supra)colloidal structures using tectonic subunits. As a consequence, a bottom-up edification is allowed from tectons into (supra)colloidal particles with higher degrees of organization (Graphical Abstract). These (supra)colloidal systems can be very useful to obtain catalysts with tunable amphiphilic properties. In this perspective, an overview of colloidal tectonics concept is presented as well as its use for the design of new, smart, and flexible catalytic systems. Finally, the advantages of these catalytic devices are discussed and the perspective of future developments is addressed especially in the context of “green chemistry.”

## Introduction

In the 1960s, Pedersen, Lehn and Cram (1987 Nobel Prize) developed the principles of supramolecular chemistry based on the molecular subunits self-assembly through non-covalent interactions (James, 2017). This self-assembly allows the construction of large structures (micelles, vesicles…) but it is also important to crystal engineering. Crystalline molecular networks can be formed by the self-assembly of molecules containing complementary recognition sites and by using a specific assembling algorithm (Hosseini, 2004). In 1991, Simard et al. (1991) proposed to call “*tectons*” these molecules with strong and well-defined attractive forces that induce controlled and predictable molecular networks in the solid state. Therefore, molecular tectonics is “*the art and science of supramolecular construction using tectonic subunits*” (Su et al., 1995) and it is based on molecular recognition events and their iteration (Hosseini, 2005).

**Graphical Abstract:**
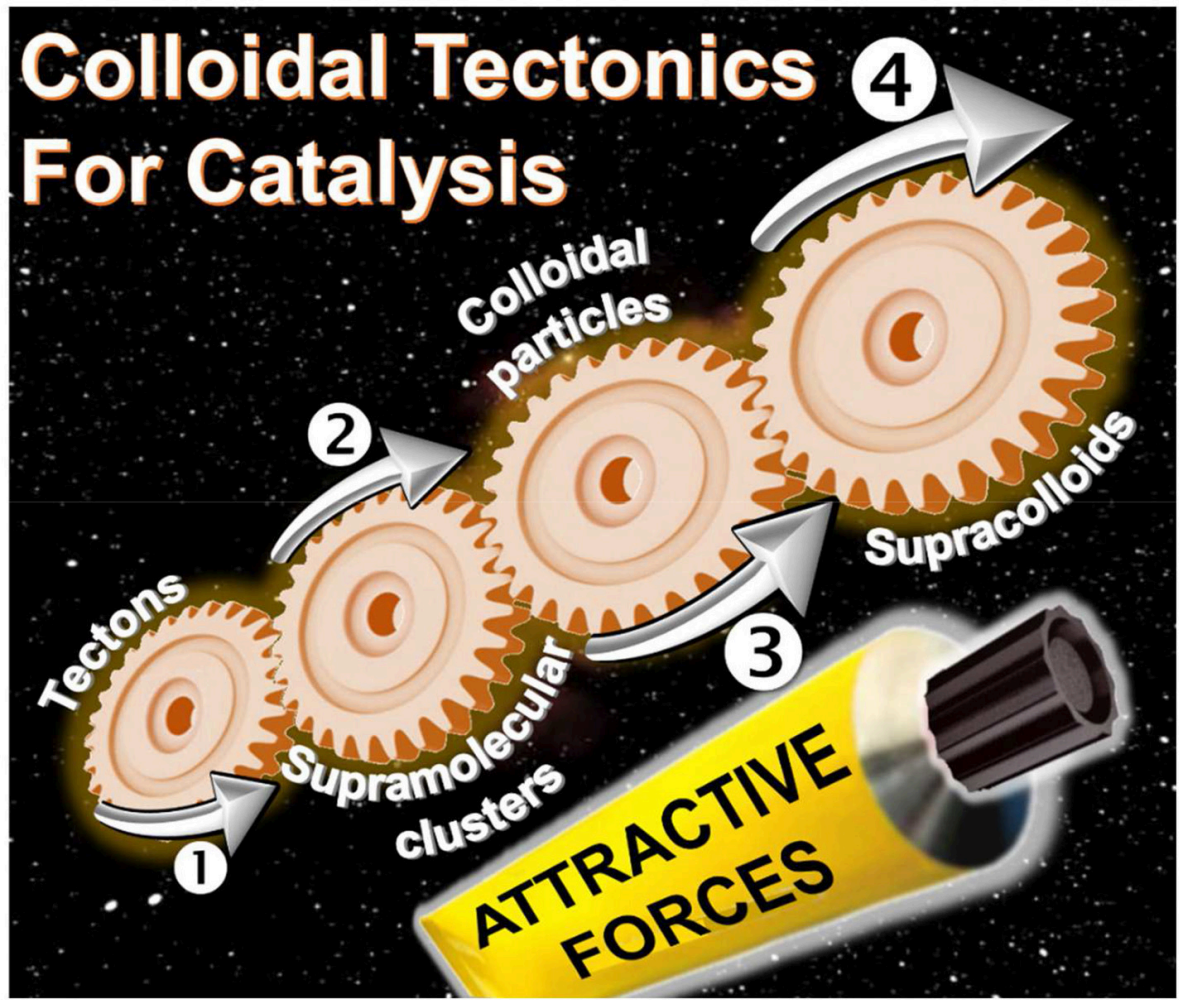
Bottom-up edification of colloidal or supracolloidal systems from tectons.

On the other hand, particles can be used as building blocks to obtain complex superstructures. In 2003, the term of “*colloidal molecules*” has been introduced to describe structures made by the self-assembly of particles under the effect of attractive forces and/or external environmental effects (van Blaaderen, 2003). Their synthesis requires the formation and the drying of Pickering emulsions (i.e., emulsions stabilized by particles) (Nanoharan et al., 2003). It is noteworthy that the interactions of particles at interfaces are also suitable templates to arrange particles into colloidal

crystals (Pieranski, 1980), colloidosomes (Velev et al., 1996; Dinsmore et al., 2002), etc. All these objects are supracolloidal assemblies in the strict sense of that term, since they emerge from the packing of particles (Li et al., 2011). This concept includes Pickering emulsions as their formations and stabilities depend not only on the particles size but also on particle-particle interactions and, of course, particle-water and particle-oil interactions (Binks and Clint, 2002; Boker et al., 2007). These Pickering emulsifiers can be very useful to replace generally harmful and unsafe molecular petro-sourced surfactants. One of the most widely used particles with emulsifying property is probably silica. As native silica is too hydrophilic to ensure good stabilization of emulsions (i.e., they remain dispersed in the polar phase), silica is “reprogrammed” through the introduction of hydrophobic binding sites leading to their interfacial interlocking thereby enhancing stability (Yang et al., 2017a).

**Figure 1:**
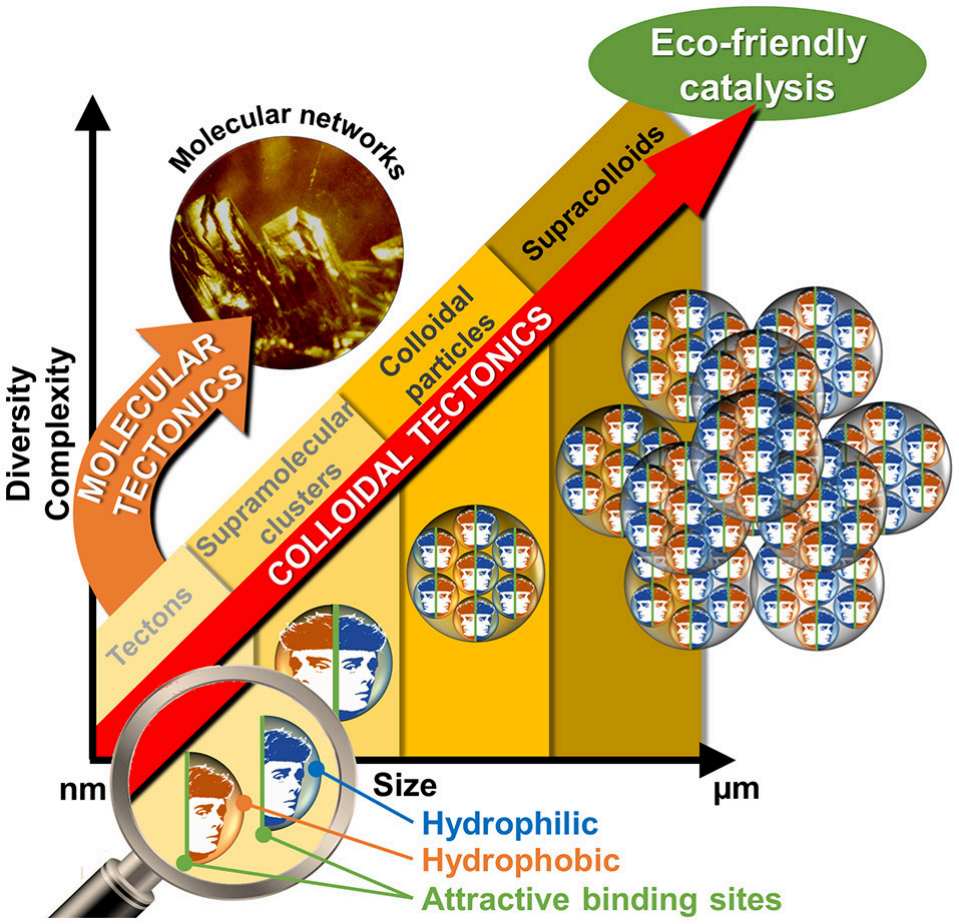
From tectons to molecular and colloidal engineering of nano- and microcatalytic systems.

If, the interactions between molecules or particles can be used to obtain a variety of self-assembled systems named supramolecules or supracolloids, it is possible to combine these two fields in order to obtain iterative self-assembling processes from molecules into (supra)colloidal particles. By analogy with the molecular tectonics definition, I propose to use “colloidal tectonics” to define the art and science of supramolecular formation of (supra)colloidal structures using tectonic subunits (molecular building blocks). Consequently, a bottom-up construction of large colloidal systems (until microscale) is allowed from tectons (Leclercq et al., 2015). The most important aspect of this method appears to be the fact that the assembling process is based on molecular recognition operating at the level of the complementary tectons providing an infinite variety of reversible (supra)colloidal systems with predictable, versatile and switchable properties. As a result, these nanostructured systems can be useful for a vast array of topics including drug delivery or catalytic facilitation systems (Yang et al., 2017b). If the use of multiphasic systems (solid/liquid, S/L, liquid/solid/liquid, L/S/L, etc.) is a smart solution to favor the separation of reaction products and the catalyst recovery, the mass transfer clearly limits the reaction rate. To solve this issue, catalytic (supra)colloidal systems (colloidal suspensions, Pickering emulsions, etc.) can be very useful as they exhibit several advantages: (i) presence of immiscible particles, optionally, with catalytic activities, (ii) improvement of mass transfer ascribed to a huge interfacial contact, (iii) easy separation of the reaction products facilitating the catalyst recovery, (iv) upgrade of the overall ecological aspect of the process (Pera-Titus et al., 2015). However, the “programming” of particles to make them amphiphilic often requires harmful synthetic methods. In order to obtain more environmental benefits with these systems, the bottom-up synthesis of (supra)colloidal systems by colloidal tectonics constitutes a novel and versatile field with promising green credentials for catalytic reactions (Figure 1). The goal of this perspective is to illustrate the foundations of these systems that can be involved in various catalytic processes. As an interdisciplinary researches, I posit concise, comprehensive schemes for the design of these new, smart, and switchable catalytic (supra)colloidal systems that reflect recent advances in this new topic. Finally, I put forward future research directions and issues to be considered especially in the context of “green chemistry.”

## From milk to colloidal tectonics concept

As is often the case, the vastness and complexity of nature offers numerous motivation and inspiration for scientists. Consequently, many synthetic supramolecular systems are designed to copy functions of ingenious biological systems (Wilson and Jayawickramarajah, 2017). Colloidal tectonics does not escape this reality because nature already uses this concept to obtain supramolecular colloidal structures.

### Natural systems

**Figure 2:**
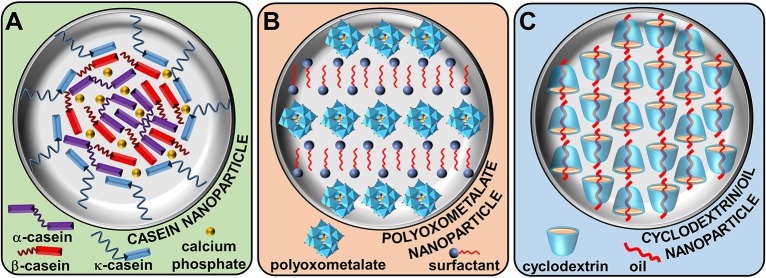
Natural **(A)** and artificial **(B,C)** supramolecular colloidal systems based on colloidal tectonics approach.

Milk is an extraordinarily complex and physically stable water-based fluid, able to vectorize proteins, lipids, and calcium phosphate which is the main source of nutrition for infant mammals (Horne, 2002). At the heart of this biological fluid, we find four phosphoproteins: α_S1_, α_S2_, β, and κ-caseins (Huppertz, 2013). These peptides contain a high number of glutamic acid, proline, leucine, and lysine (ranked in order of importance, Park, 2011). As caseins are non-crystallizable proteins, their folding are only partially known. However, since proline acts as a structural disruptor due to its rigidity, a particular bending of the caseins chain is observed leading to the inhibition of close-packed and ordered 3D structures. In contrast, the block distribution of hydrophobic (leucine) and hydrophilic amino acids (lysine and glutamic acid) confers to caseins an amphiphilic nature. Nevertheless, since the lack of tertiary structure, there is a considerable exposure of the hydrophobic residues leading to a strong self-association of caseins in aqueous solution to form supramolecular assemblies called casein micelles. It is noteworthy that casein micelles show only superficial resemblance to surfactant micelles. Indeed, casein micelles are large spherical water-insoluble aggregates (size from 20 to 600 nm in diameter) composed of several thousand associated casein subunits. The most hydrophilic protein (i.e., κ-casein) resides at the surface whereas the most hydrophobic ones are located inside of the micelle (Figure 2A).

The casein micelles growth is controlled by the steric repulsions of κ-caseins (Holt and Horne, 1996). In addition, the electrostatic repulsion increase could be a contributory factor (Horne, 2002). However, in sharp contrast to surfactant micelles, the interior of casein supramolecules remains highly hydrated: they are porous structures. In addition to these hydrophobic forces, casein proteins are able to interact with colloidal calcium phosphate through electrostatic forces (McMahon and Oommen, 2013). Although the casein micelles are in dynamic equilibrium with their environment and that the structural arrangement can be modified by environmental changes (pH, temperature, etc.), the hydrophobic and electrostatic forces prevent spatial changes and dissociation of the supramolecular structure. Casein micelles can be seen as open sponge-like colloidal structures that respect the principles of self-aggregation, interdependence, and diversity observed in nature (McMahon and Oommen, 2013). On the other hand, casein micelles can adsorb at the water/oil interface leading to Pickering-like emulsion (Dickinson, 2010). Indeed, casein micelles cannot reasonably be considered as classical Pickering emulsifiers (e.g., inorganic nanoparticles) due to their deformable supramolecular texture. Nevertheless, this biological example is very instructive as the spontaneous bottom-up formation of (supra)colloidal structures using tectonic subunits (i.e., colloidal tectonics) can be envisaged from a synthetic point of view. Firstly, the essential driving force leading to casein micelles (colloidal particles) formation is clearly the interactions between the hydrophobic regions of the caseins. Secondly, the colloidal supramolecules growth is inhibited by steric and electrostatic repulsions. Thirdly, this self-aggregation process results in a porous colloidal particles constituted of hydrophilic and hydrophobic regions in which solvent(s) can be accommodated. Fourth, there is a close relation between the structure of the caseins (tectons) and the physicochemical properties of the resulting particles. Indeed, the particles association in water/oil biphasic system induces the formation of Pickering emulsions in which each particle is positioned predictably with respect to its “hydrophilic/hydrophobic balance.” As a consequence, a continuously transition of the properties is effected between the tectons, the particles structures and the properties of the resulting Pickering emulsions.

### Artificial systems

To obtain the spontaneous formation of (supra)colloidal structures using tectonic subunits, it is necessary to keep in mind that the total interaction energy can be considered to be the sum of hydrophobic attraction, steric and electrostatic repulsions. To achieve colloidal structures stabilized by hydrophobic/hydrophilic intermolecular forces, it is necessary to use building blocks (tectons) with precise and scalable algorithm. A simple strategy is to self-assemble two tectons with opposite polarities using complementary binding sites leading to stable discrete supramolecular clusters. Next, the hydrophobic effect will be responsible for the clusters self-assembly into (supra)colloidal structures (Figures 1, 2).

The first well-investigated system was published in 2012. This system results from ionic metathesis between anionic polyoxometalates (POMs) and cationic surfactants (“hydrophilic” and “hydrophobic” tectons) leading to the formation of uncharged clusters (Leclercq et al., 2012). In aqueous solution, clusters form spontaneously nanoparticles in order to decrease the hydrophobic/water contact. These particles contain parallel inorganic planes of unconnected POMs, separated by interdigitated cationic surfactant chains (Figure 2B). It is noteworthy that this organization is highly predictable because it is typical of ionic surfactants in solid state. Even if self-assembly is governed by the normal processes of nucleation and growth, defect regions appear in the lamellar organization and lead to the appearance of surface charges that limit the particle growth (Leclercq et al., 2014). According to the POM nature, the size or the shape of the nanoparticles can be modified from spherical to needle-like structures (Leclercq et al., 2015). Nevertheless, the lamellar structure is completely independent of the size or the shape of the POMs as it results from the combined effects of hydrophobic, van der Waals, electrostatic, and steric forces enable tectons to arrange themselves. This internal structure results in a porous particles in which small organic molecules can be accommodated (Leclercq et al., 2014). Consequently, in the presence of water and aromatic oil, these particles can be used as surface-active building blocks for the preparation of very stable water-in-oil Pickering emulsions consisting of shell-like microarchitectures that exhibit a high cohesiveness between the particles located in the interfacial layer. Indeed, the penetration of oil molecules into the particles results in the release of some alkyl chains allowing their interlocking and the increase of the interfacial elasticity. This property offers a general route to the construction of various supracolloidal structures such as colloidosomes (Leclercq et al., 2014).

The second system is based on cyclodextrines (CDs). Although the stabilizing effect of CDs on biphasic oil/water systems is known since the 1990s (Shimada et al., 1991), it is now clear that this mechanism comes from the partial wettability of the surface-active insoluble CD/oil inclusion complexes by the two phases leading to oil-in-water Pickering emulsions (Hashizaki et al., 2007). However, the nature of the precipitated complexes remains debated. Some authors have claimed that microcrystals (Davarpanah and Vahabzadeh, 2012) stabilize the droplets whereas others propose the formation of spherical nanoparticles (Leclercq et al., 2013). In fact, these both assertions are good since the colloidal structure is concentration-dependent: spherical nanoparticles at low CD concentrations and microcrystals for greater ones (Leclercq and Nardello-Rataj, 2016). Therefore, these systems result from host-guest interactions between CDs and oil (“hydrophilic” and “hydrophobic” tectons) leading to the formation of inclusion complexes (discrete supramolecular clusters). These clusters self-assemble spontaneously by the normal processes of nucleation and growth to form nanoparticles. By analogy with the crystalline structures of host-guest complexes, it is reasonable to assume that these particles contain a channel-type structure through the formation of H-bond network between the hydroxyl groups of CDs (Noltemeyer and Saenger, 1980; Sicard-Roselli et al., 2001; Gallois-Montbrun et al., 2013). The guests are embedded inside the tubular cavity of the CD columns (Figure 2C). The channels adopt a close packing leading to the incorporation of interstitial water molecules. Unfortunately, in contrast to well-optimized growth conditions, the interfacial rigidity increases with the particles emergence leading to slower CD or oil transfer rates across the L/S/L interface limiting their growth. Obviously, in addition to Pickering emulsions, these particles lead to the formation of various other supracolloidal structures such as colloidal crystals (Leclercq and Nardello-Rataj, 2016), colloidosomes (Mathapa and Paunov, 2013), etc. It is noteworthy that the guest can also be a polymer (Potier et al., 2013). In this case, columnar CD domains (crystallites) acting as physical cross-links between the polymer chains not included leading to the formation of supramolecular hydrogels and providing a good platform to obtain Pickering-like emulsions. However, the growth rate is limited by the diffusion, i.e., by the rate at which the CDs are transfer from the aqueous bulk to the growing crystallites. As the hydrogels are obtained after heating/cooling cycle, a better crystallization is observed compared to the previous systems.

## Catalytic applications

Throughout the section above, I have presented colloidal tectonics that enables the smart design of nanoparticles with tunable amphiphilic properties that can promote the catalytic activity of multiphasic systems *via* the formation of stable S/L or L/S/L dispersions. Following this concept, new catalytic platforms based on (supra)colloidal systems are obtained.

### S/L nanocatalytic colloidal systems

In 2014, our group reported the use of amphiphilic catalytic dodecyltrimethylammonium/PW_12_O_40_ nanoparticles to perform olefin epoxidation in eco-friendly solvents (Mouret et al., 2014). Due to its amphiphilic properties, the self-assembled particles are able to form S/L dispersions with cyclopentyl methyl ether, 2-methyl tetrahydrofuran, methyl acetate, and glycerol triacetate. With these four solvents, the epoxidation of cyclooctene proceeds at competitive rates (TOF_0_ > 260 h^−1^), good yields (>95%) and high selectivity (>99%). It is noteworthy that the catalytic activities are directly correlated to the dispersion stability in a given solvent: the better the stability the higher the activity. Moreover, the self-assembled nanoparticles are much more active than the native POM (TOF_0_ × 10). This effect can be clearly related to the localization of the catalyst in the interfacial layer as well as the accommodation of substrates inside the porous nanoparticles. Such catalytic systems clearly combine the advantages of homogeneous and heterogeneous catalysis: high activity and selectivity, ease of phase separation, re-use of catalyst (after filtration and distillation).

### L/S/L microcatalytic supracolloidal systems

These systems are actually the so-called Pickering emulsions which are good platforms to enhance mass transfer between substrates with opposite polarity (see above). Two kind of processes can be used: “Pickering-Assisted Catalysis” (PAC) and “Pickering Interfacial Catalysis” (PIC, Pera-Titus et al., 2015). The PAC system constitutes the simplest approach in which a homogeneous catalyst is combined with Pickering emulsifiers. In contrast, the PIC system uses particles behaving concomitantly as emulsifiers and interfacial catalysts.

In 2012, the dodecyltrimethylammonium/PW_12_O_40_ nanoparticles are used to perform olefin epoxidation in water/toluene biphasic system using H_2_O_2_ as oxidant (Leclercq et al., 2012). The quantitative cyclooctene, cyclohexene and limonene epoxidation could be achieved with easy product and catalyst separation. It is noteworthy that the *in situ* ionic metathesis between POM and dodecyltrimethylammonium (molar ratio 1:3) provide a smaller catalytic activity than the system made with the preformed nanoparticles (TOF_0_ divided by a factor three). This behavior can be ascribed to a partial ionic exchange providing a mixture of supramolecular clusters that contribute to the apparition of defects in the lamellar packing limiting the particle growth (see the previous section). In these conditions, the emulsions are rapidly destabilized into biphasic media leading to inefficient catalytic systems. Therefore, the neat correlation between the catalytic performance and emulsification of biphasic systems results clearly from a PIC effect providing a much larger water/oil interfacial area where the catalytic nanoparticles are localized. Moreover, it is relevant to note that these catalytic performances are obtained under mild conditions (65°C) and without stirring (except during the emulsification) indicating that the process is only driven by the catalytic cycle.

In 2013, two research teams independently prospected the catalytic applications of Pickering emulsions based on CD inclusion complexes combined with homogeneous catalysts (i.e., PAC systems). One is based on the extemporaneously formation and adsorption of CD/oil insoluble complexes at the water/oil interface without any synthesis (see above). This system has been used for the oxidation of olefins, organosulfurs, and alcohols using [Na]_3_[PW_12_O_40_] as water-soluble catalyst and H_2_O_2_ as oxidant (Leclercq et al., 2013). These Pickering emulsions behave as a highly efficient reaction medium. For instance, the epoxidation of cyclooctene in water/heptane biphasic system proceeds at competitive rate (370 h^−1^), good yield (>99% in 30 min) and high selectivity (>99%) due to the promoted interfacial contact between the substrate and the catalyst. Such catalytic emulsion allows straightforward separation of the phases by centrifugation or by heating. In addition, these systems can be used without any organic solvents for liquid substrates. The other uses the formation of inclusion complexes between CDs and polyethylene glycol (PEG) leading to the formation of hydrogels that providing a good platform to obtain Pickering emulsions (see above). Rhodium-catalyzed hydroformylation of higher olefins, at commercially competitive rates, can be successfully performed in these emulsions (Potier et al., 2013). Indeed, the conversion of 1-decene was 16-fold higher than that observed in neat water and 4-fold higher than that measured using PEG as sole additive. In addition, the regioselectivity (linear to branched aldehyde ratio) was constant. Therefore the equilibrium between the catalytic species is not significantly modify and the mass transfer improvement is only due to the contact area increase between the olefins and the water-soluble catalyst.

## Conclusion and perspectives

**Figure 3:**
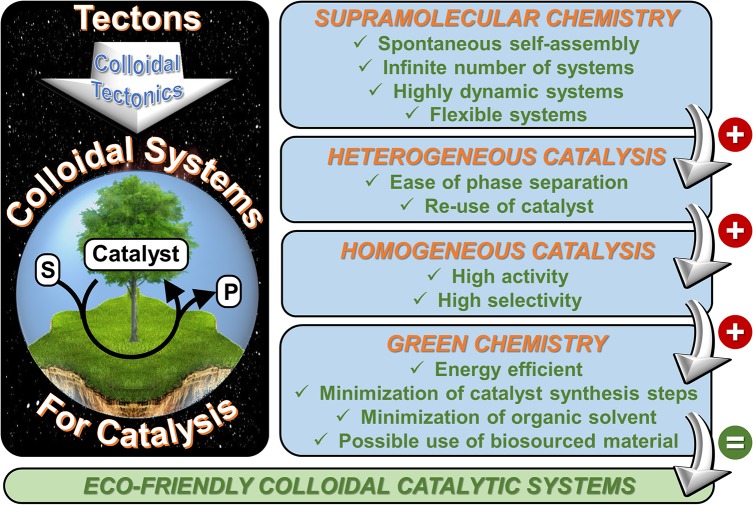
Main benefits for the use of colloidal tectonics in the engineering of nano- and microcatalytic systems.

The present manuscript develops a new approach that lies at the crossroads between supramolecular and colloidal chemistry which is called “*colloidal tectonics*.” The foundations, design, synthesis, and structure of these systems are illustrated with the aid of recent literature. The unifying goal is to learn how to use attractive forces to control molecular self-assembly and produce new colloidal systems with predetermined functions and/or properties closely related to the desired applications. This approach is clearly highly interdisciplinary as the scope of this research is well beyond the traditional frontiers of organic chemistry. This methodology covers a wide field of investigation which could be applied in many domains such as cosmetics, pharmaceutical formulations, nanomaterials, catalysis, etc. In the case of the rational design of catalytic systems, the colloidal tectonics approach is highly compatible with some concepts of “green chemistry” (Figure 3). Moreover, this allows the formation of intermediate systems between homogeneous and heterogeneous mixtures from macroscopic and microscopic point of view, respectively, thus combining the properties of homogeneous and heterogeneous catalysis. Consequently, the water/oil interface greatly favors the mass transfer between the substrate(s) and the catalyst: this system works without stirring (Pera-Titus et al., 2015). On the other hand, the compartmentalization of reactants and products avoids or limits the side reactions (Leclercq et al., 2012).

It is noteworthy that the colloidal tectonics approach is closely related to the phase-boundary catalysis (Nur et al., 2000, 2001, 2004). Indeed, these catalytic systems also combine the advantages of reactions performed in the interfacial area since the catalytic active sites are located on the external surface of amphiphilic zeolite particles (i.e., at a phase boundary). The major difference between these catalytic systems is related to the catalyst preparation. Indeed, synthetic steps are required for the phase-boundary catalysis whereas colloidal tectonics approach uses self-assembly “bottom-up” processes (Ikeda et al., 2001). Nevertheless, the self-assembled particles must be stable in solvents for catalytic applications (i.e., tecton-tecton, supramolecular cluster-supramolecular cluster and particle-particle interactions must be higher than tecton-solvent, supramolecular cluster-solvent, and particle-solvent interactions). Consequently, it will not be easy to found good tectons for that. However, in the light of the scientific data available, the following empirical guidelines can be formulated: (i) at least two molecular tectons with opposite polarities (i.e., polar and apolar) can be used, (ii) a restricted conformational space (i.e., rigid structure) is required for at least one tecton (e.g., POM, CDs) whereas the other must be flexible (e.g., alkanes, alcohols), and (iii) the tectons are capable of mutual interactions (molecular recognition) in order to form discrete supramolecular clusters. Against this background, the clusters can be self-assemble, through solvophobic effect into colloidal particles. The exciting prospects of these new catalytic systems will provide a strong impetus for researchers. As supramolecular chemistry knows no limit except that of our imagination, this approach creates a particular exciting environment for research in molecular and colloidal sciences. Therefore, this new methodology is an invitation to creativity and innovative capacity that can trigger movement, action, and change in the face of environmental problems.

## Author contributions

The author confirms being the sole contributor of this work and approved it for publication.

### Conflict of interest statement

The author declares that the research was conducted in the absence of any commercial or financial relationships that could be construed as a potential conflict of interest.
